# Alcohol use in Australia: countering harm with healing

**DOI:** 10.1016/j.lanwpc.2023.100774

**Published:** 2023-05-17

**Authors:** Lorelle Holland, Natasha Reid, Nicole Hewlett, Maree Toombs, Tylissa Elisara, Amy Thomson, Tracy Humphrey, Andrew Smirnov

**Affiliations:** a School of Nursing, Midwifery and Social Work, The Faculty of Health and Behavioural Sciences, The University of Queensland, St Lucia, Queensland 4006, Australia; b Child Health Research Centre, Faculty of Medicine, The University of Queensland, 62 Graham Street, South Brisbane, Queensland 4101, Australia; c School of Public Health, Faculty of Medicine and Health, The University of Sydney, Fisher Road, Camperdown, New South Wales 2006, Australia; d School of Education, Faculty of Humanities and Social Sciences, The University of Queensland, St Lucia, Queensland 4006, Australia; e Faculty of Health and Behavioural Sciences, The University of South Australia, 101 Currie St, Adelaide 5000, Australia; f School of Public Health, Faculty of Medicine, The University of Queensland, Herston, Queensland 4006, Australia

**Keywords:** Australia, Alcohol, Healing, Culture, Public health, Community, Best-practice, Harm minimisation, Colonisation, Indigenous health, Social and emotional wellbeing, Trauma, Global health

## Abstract

Harmful use of alcohol consumption in Australia is a serious socio-political and public health issue that is exacerbated by exploitative marketing campaigns by the alcohol industry. In Indigenous populations harmful alcohol use is directly related to the legacy of colonisation that has led to complex social issues and adverse intergenerational trauma. To effectively address alcohol-related harm in Australia, it is necessary to critically apply the ‘Three Pillars of Harm Minimisation’, which are demand reduction, supply reduction, and harm reduction. This can be facilitated through approaches such as the ‘Interplay Wellbeing Framework’, which situates concepts of wellbeing and risky alcohol use within the context of systemic inequities across all social determinants of health. Culturally responsive approaches embody a holistic view of community, mutually respectful collaboration, culture, healing, and self-determined change. This is underpinned by Indigenous leadership that promotes existing resistance, resilience, interpersonal relationships, and strengths that instil healing to counter the harms associated with alcohol use.

## Background

Australia is a wealthy nation with a high standard of living and well-resourced health, educational, social, and welfare systems.^1^^,^^2^ However, for many Aboriginal and Torres Strait Islander people, respectfully referred to as Indigenous Australians henceforth, who collectively make up 3.3% of the Australian adult population,^3^ are exposed to systemic inequities across all social determinants of health (e.g., housing, education, and health service access).^4^^,^^5^ ‘In 2018, the gap in health-adjusted life expectancy at birth between Indigenous and non-Indigenous Australians was 15.5 years for males and 14.7 years for females’.^6^ ^(pvii)^ Inequities are a direct result of widespread harm, oppression, subjugation, and racially discriminatory government practices applied during the last 235 years of colonial occupation and rule.^4^^,^^5^^,^^7^^,^^8^ Colonisation and the arrival of migrants during the 18th century brought with it the supply of potent alcohol and a culture of drunkenness, which were foreign to Indigenous Australians prior to colonisation.^9^ The enduring negative consequences of colonisation and alcohol-related harm continues to contribute to a preventable yet significant 4.5% of the 38% total disease burden in Australia today.^10^ Unequal health outcomes associated with alcohol consumption contributes to 10.5% of the preventable total disease burden experienced by Indigenous Australians.^6^ Whilst Indigenous Australians choose to abstain from alcohol consumption at a higher rate than non-Indigenous Australians at 31% compared with 23%, respectively,^11^ rates of risky alcohol consumption for Indigenous Australians aged 15 years and over are 18.7% compared with 15.2% for non-Indigenous Australians.^12^ To address complex social issues that drive harmful levels of alcohol consumption by Indigenous Australians it is necessary to centre Indigenous-led and self-determining strategies from an Indigenous standpoint.

## Indigenous standpoint

**Fig. 1:**
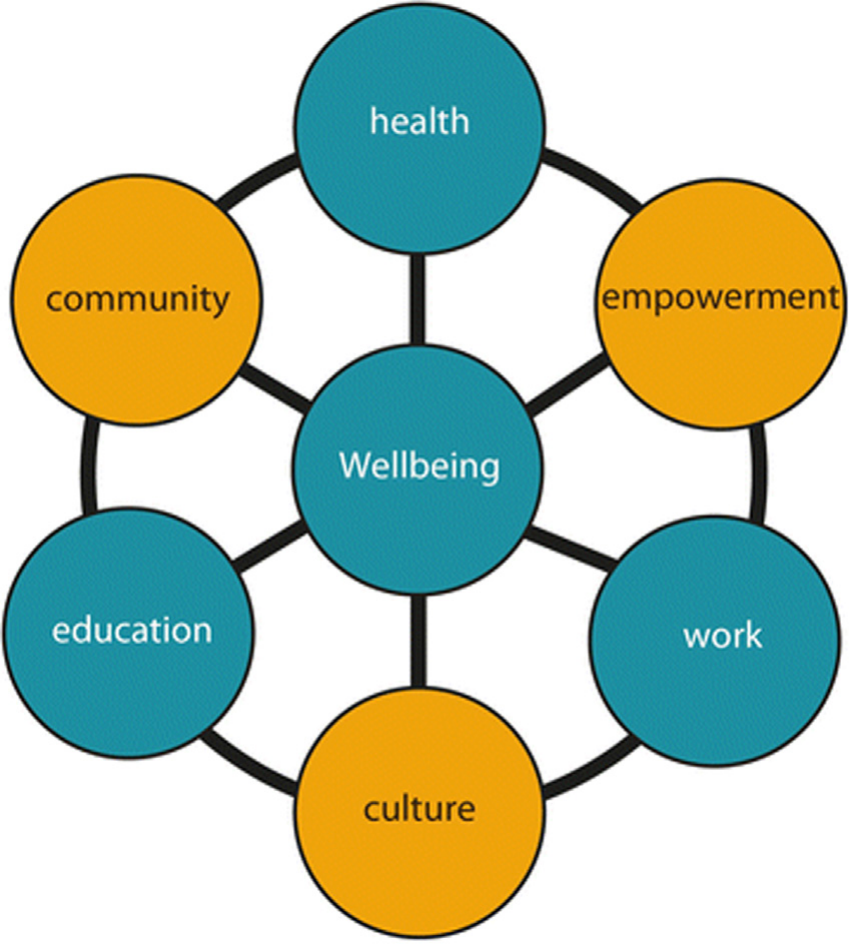
Interplay wellbeing framework.^24^^(p4)^

From an Indigenous standpoint, culturally responsive initiatives that embed strengths-based public health approaches can direct health policy, promoting a shift towards healing by prioritising culture, strength, resistance, and resilience and ultimately reducing the entrenched deficit discourse.^13^ Deficit discourse wrongfully permeates government health policy when patriarchal white authority cultivates racist stereotypes of Indigenous Australians by depicting an identity of disadvantage and dysfunction.^14^, ^15^, ^16^, ^17^ The application of problematising Indigeneity in this way fails to recognise racism as a determinant of health, which drives ongoing disparities and inhibits self-determination strategies to address alcohol-related harm.^7^^,^^8^^,^^17^, ^18^, ^19^, ^20^ Indigenous rights and self-determination principles are embraced within this work by centering sovereign Indigenous authorship and world-views, which respect Indigenous ways of knowing, being and doing.^19^^,^^21^, ^22^, ^23^ To counter racism, the process of truth telling hopes to enact a contemporary path to healing, which recognises intergenerational trauma and related risky alcohol use as a direct result of the negative impacts of colonisation. The current commentary provides a critical overview of alcohol-related harm in Australia and culturally responsive residential services guided by the ‘Interplay Wellbeing Framework’ (see Fig. 1).^24^ This framework builds upon Aboriginal concepts of wellbeing to create a novel, inclusive, and collaborative methodology that shifts the focus towards healing and counters deficit discourses.^24^ The framework was developed using a collaborative methodology called the ‘shared space’ in which the government, research, and Aboriginal community partners were on an equal footing in all project stages, and the work was underpinned by well-developed relationships.^25^ The framework brings together *Aboriginal-identified holistic wellbeing values of culture, empowerment, and community* with Australian Government ‘Closing the Gap’ priorities of education, employment, and health.^24^ The Interplay Wellbeing Framework is used to measure these domains and was developed in remote communities where a significant relationship between reduced alcohol consumption and improved outcomes across culture, education, and employment was identified.^24^

Alcohol policy in Australia is guided by the National Drug Strategy^26^ (the strategy) and relevant sub-strategies, including the National Alcohol Strategy (2019–2028)^11^ and the National Aboriginal Torres Strait Islander Peoples Drug Strategy (2014–2019).^27^ These strategies are underpinned by a harm minimisation approach, comprising the ‘three pillars’ of supply reduction (e.g., alcohol availability and policing), demand reduction (e.g., prevention of alcohol uptake and delay of onset of consumption, education, and treatment), and harm reduction (e.g., responsible service of alcohol and sobering-up shelters).^26^ Critics of the implementation of the strategy have contended that the allocation of resourcing is too strongly weighted toward supply reduction, especially law enforcement, with inadequate funding of demand reduction and harm reduction activities.^28^ The most recent iteration of the strategy, the National Drug Strategy 2017–2026,^26^ highlights the need for stronger participatory processes and community engagement, alongside a continued commitment to evidence-based activities, including the monitoring and evaluation of funded programs. Alongside this, the National Aboriginal Torres Strait Islander Peoples Drug Strategy^27^ is rightly guided by principles that include ‘Aboriginal and Torres Strait Islander ownership of solutions’ ^(p4)^ and ‘holistic approaches that are culturally safe, competent and respectful’.^(p17)^

Approaches such as the Interplay Wellbeing Framework show how government and communities could work together in a shared and respectful space to progress the National Drug Strategy. It is necessary to develop appropriate initiatives to reduce alcohol-related harm for Aboriginal and Torres Strait Islander communities, which can be evaluated against value-based domains such as those of culture, empowerment, and community.

## Impacts of colonisation

Intergenerational trauma manifests as poor psychological wellbeing and is interlinked with risky alcohol consumption for some Indigenous Australians in contemporary Australia.^29^^,^^30^ Indigenous Australians lived in good holistic health for 40–60,000 years prior to the introduction of a potent alcohol supply.^9^ Within colonial enforced dominance, many human rights violations occurred such as genocide, massacres, and enslavement.^5^^,^^31^ Furthermore, land was dispossessed, and ecological habitats destroyed, culture was disregarded and punished, spiritual beliefs, identity, and values were deprived, languages denied, and cooperative family kinships were disrupted by discriminatory and racist policies, including the systemic removal of Indigenous Australian children from their families, which became known as the ‘Stolen Generations’.^5^^,^^31^^,^^32^ These impacts of colonisation continue to cause high levels of stress and trauma for Indigenous Australians today.^5^^,^^33^ Exposure to dehumanising mistreatment as a direct result of colonisation and ongoing structural racism continues to negatively impact upon Indigenous Australians wellbeing and socio-economic status, which are directly linked with higher levels of alcohol consumption.^29^ Consequently, harm minimisation strategies need to not only address the social determinants of health but ways to heal the suffering that is leading people to seek solace in alcohol use.^29^

## Unequal alcohol-related harm

The Northern Territory (NT) has the lowest population of people within any state or territory in Australia and has a tenfold proportion of Indigenous Australians.^34^ Significant research concerning harmful alcohol use has focused on the Northern Territory (NT) in Australia as there is a per capita alcohol consumption that is 1.3–2.0 times higher than the national average.^35^^,^^36^ Alcohol sales and the connection with health care utilisation and deaths in the NT is further evidence of the ongoing challenges faced by these communities. In the NT, age-standardised alcohol-attributable death rates are 3.5 times greater and alcohol-related hospitalisation rates are more than double the national rate.^35^^,^^36^ Globally, government strategies to reduce alcohol-related harm have included a minimum drinking age, penalties for drink driving, alcohol warning labels, licencing, and taxation policies.^37^ However, there are inconsistencies in the way these strategies are implemented in Australia (e.g., differential taxation rates that ensure the availability of cheap alcohol products), which are a consequence of industry influence.^38^ Furthermore, the effectiveness of these strategies may be limited if they are not accompanied by approaches that address wellbeing factors, including those of culture, empowerment, and community. Consequently, Indigenous Australians’ communities continue to be impacted unequally by alcohol-related chronic disease, disability, and premature deaths.^37^

## The alcohol industry: a barrier to effective demand reduction strategies

The public health message of harm associated with exposure to alcohol consumption in Australia is diluted by a deliberate attempt by the alcohol industry to control health warnings.^39^ The ‘DrinkWise’ program (funded by the alcohol industry) seeks to ‘blame the consumer’ as an irresponsible drinker; this reduces the need for the alcohol industry to take responsibility for their harmful products and to fund effective harm minimisation strategies.^39^^,^^40^ This approach can only be described as exploitative within the context of serious injury, violence, illness, and disease associated with alcohol use.^12^^,^^39^^,^^41^, ^42^, ^43^ Further complicating the situation, the alcohol industry has capitalised on the challenges communities face by providing easy access to alcohol through the high density development of alcohol outlets in urban lower socio-economic status areas.^44^ This exploitative practice poses an increased risk of alcohol-related harm to people living in these areas.^44^

## Imposed supply reduction strategies and barriers

In Australia, the legal age to purchase and consume alcohol is 18 years and consumption is largely unrestrained due to social acceptance, which includes overt alcohol advertising and marketing.^41^ There are some limited formal constraints on alcohol consumption in the form of varying laws and alcohol licencing restrictions in different Australian states and territories, which dictate where and when alcohol can be consumed and purchased.^45^ For Indigenous Australians, depending on location, further restrictions can apply.^46^ There are many communities in Australia that alcohol prohibition regulations and management laws are enforced.^47^, ^48^, ^49^ Whether they are self-designated or designated by government, some Indigenous Australian communities are ‘dry areas,’ meaning that the consumption or possession of alcohol is prohibited.^45^ Further alcohol restrictions in some communities include limitations regarding the time of day and the strength and volume of alcohol that can be purchased.^46^ During COVID 19, to maintain social distancing, a temporary relaxation of liquor licencing restrictions occurred in every state and territory except the Northern Territory; this saw an increase in alcohol consumption as the sale of alcohol was permitted as online orders including home delivery from restaurants and alcohol outlets.^50^

## Understanding the need for harm reduction

The National Alcohol Strategy 2019–2028 outlines that many people who drink at risky levels do not consider themselves as heavy drinkers and nor do they attribute alcohol use as a cause of ‘cancer, cerebrovascular, cardio-vascular, liver and digestive disease’.^11^ ^(p6)^ To counter widespread social acceptance of alcohol consumption in Australian society in addition to a reduced awareness of alcohol-related harm, harm reduction strategies are paramount throughout all stages of the life-span. For example, a recent harm reduction campaign was launched by The Foundation for Alcohol Research and Education (FARE)–the National Awareness Campaign for Pregnancy and Breastfeeding Women (the Campaign) from July 2020 to September 2024.^51^ The campaign aims to help prevent prenatal alcohol use in Australia, as research indicates 1 in 2 women report consuming alcohol during their pregnancy and 1 in 4 women consumed alcohol following awareness of their pregnancy.^11^ Prenatal alcohol use can lead to a range of birth defects and neurodevelopmental impairments, and for some people can lead to a lifelong disability known as fetal alcohol spectrum disorder (FASD).^11^

Harm reduction strategies are also necessary to prevent the 1452 premature deaths that were attributed to alcohol in 2020; 396 were female and 1056 were male deaths.^12^ Furthermore, in 2020, alcohol-related hospitalisations accounted for 86,400 admissions, and contributed to 12% of the health gap between Indigenous and non-Indigenous Australians. The interrelated features of alcohol-related harm have been associated with physical assaults, injuries, child neglect and abuse, sexual crime, increased risk of suicidality, and partner violence; and ultimately lives being lost prematurely to events of family violence, homicide, suicide and accidents.^11^^,^^12^^,^^42^^,^^52^ Interpersonal violence from alcohol is the leading cause of death and injury in women aged 45-years-old or less including the murder of one woman every week in Australia.^53^ Alcohol-related harm had an estimated social cost of $66.8 billion dollars in Australia from 2017 to 2018, which included the impact on workplaces, crime, healthcare, road safety, premature death, and overall quality of life.^12^ Underlying the high rates of risky alcohol consumption for Indigenous Australians is a significant link between traumatic life experiences and common mental health challenges such as anxiety and mood disorders.^30^

## Management of alcohol in Indigenous communities

To reduce alcohol-related harm in some Indigenous Australian communities, Alcohol Management Plans (AMPs) were developed using varying and inconsistent approaches.^54^ At the outset, AMPs were intended to include a mix of supply, demand, and harm reduction measures, following an alcohol harm minimisation approach.^54^ However, in most cases, supply reduction measures such as restrictions on the availability of alcohol and increased policing in communities became the dominant features of AMPs, with limited support or resourcing for demand or harm reduction initiatives.^48^^,^^55^ This may, to some extent, reflect the politicised and ‘top-down’ approach of AMP development, which occurred despite processes that were in place to engage with communities.^56^ The lack of a balanced and community-driven approaches in these settings appeared to contribute to negative health and social outcomes, and unfortunately, there is still a lack of balance in government responses.

AMPs initially provided beneficial outcomes. An evaluation of the impacts and effectiveness of AMPs (alcohol prohibition applied in 2008) in two distinct Cape York communities showed a dramatic reduction in violence against women (Community A: 39.2% and Community B 48.2%).^53^ Additionally, survey data revealed over 75% of respondents from both communities agreed AMPs had reduced violence against women in the community.^53^ However, in some communities AMPs have resulted in a number of unintended negative consequences, such as home brewing and ‘sly grog’ practices, which can create an environment of criminality associated with possession and consumption of alcohol.^49^^,^^53^^,^^57^^,^^58^ In addition, migration out of community occurs and residents become dislocated through pursuing alcohol supplies, which displaces disruptive and violent behaviour to other localities.^59^ There has also been a lack of support for people who are alcohol dependent in communities where AMPs have been introduced.^48^^,^^55^ It is now apparent that AMPs in isolation have done little in the long-term to curb alcohol-related harm in many locations, evidenced by continued high rates of assaults, emergency department presentations, aeromedical retrievals, and intensive care unit hospitalisations.^35^^,^^53^^,^^60^^,^^61^

Reaching consensus regarding ongoing management of alcohol in Indigenous communities has been a deeply polarising issue, as seen recently in Alice Springs in the NT.^56^^,^^62^ Indigenous leaders called for and welcomed emergency restrictions on the sale of alcohol from 24 January, 2023, which resulted in an immediate decrease in alcohol-related harms including family violence and emergency department presentations, but warned these restrictions ‘should not let governments off the hook’ from addressing the underlying social determinants of alcohol-related harm.^62^

This reflects a long-standing community concern that whilst a place exists for supply restriction policies (as characterised by AMPs) to reduce alcohol-related harms, these measures alone do nothing to address underlying issues such as intergenerational trauma, poverty, housing, education, unemployment, access to alternative activities, access to adequate health care, and racial discrimination.^62^ There is further concern that AMPs are an instance of a restrictive policy not applied to all Australian citizens equally irrespective of residential location, and consequently may be viewed as racially discriminatory and politically targeted.^34^ However, Aboriginal women in many communities have strongly advocated for alcohol restrictions.^56^ For example, Aboriginal leaders June Oscar (OA) and Emily Carter and others from the remote town of Fitzroy Crossing in Western Australia’s Kimberley region, helped to create community-driven alcohol restriction policy and worked tirelessly to reduce the incidence of FASD.^63^^,^^64^ Community-led urgent action in this region was prompted in 2007 due to alcohol-related violence and crime and 55 deaths including 13 suicides. The immediate and significant benefits were evident and inspired the Lililwan Project which aimed to determine the prevalence and need of children with FASD.^63^, ^64^, ^65^ Importantly, Indigenous leaders across Australia have emphasised that alcohol restrictions alone do not address vast social disadvantages. Long-term solutions are required to heal the deep wounds that have arisen from alcohol-related violence.^66^

It is evident state and federal government policies have undermined efforts by local communities to control alcohol use, disrupting the plans of communities and exacerbating alcohol-related harms experienced by drinkers and other community members.^34^ Alcohol restriction policy needs to be driven by Aboriginal community-controlled health organisations, leaders and community members.^20^ This process needs to be authentically community-controlled, without undue interference by government or commercial entities, in a manner that aligns with principles of the UN’s Declaration of the Rights of Indigenous Peoples to self-determination.^19^^,^^20^ This may facilitate the development of policy that embodies Indigenous knowledges, including values for holistically promoting health and wellbeing.

## Healing created through culture, capacity building and community

The development of the Interplay Wellbeing Framework in remote communities found that reductions in alcohol use were linked with the strength of culture, empowerment, and community.^24^ Indigenous-led approaches that promote holistic wellbeing, such as healing through culture on Country, may be the most appropriate and effective way of alleviating Indigenous Australians shared lived experiences of intergenerational trauma and interrelated alcohol harm.^8^^,^^66^, ^67^, ^68^ Appropriate funding and resources are required for the community-level diffusion of strengths-based, healing- and trauma-informed approaches that can overcome racialised health and social systems.^66^^,^^67^ Culturally responsive healing approaches need to be evidence- and theory-based and inclusive of both traditional healing and western methodologies.^68^ Programs also need to be community-led, by local Elders, to ensure that they are community-specific and sustainable. This enables knowledge and skills to be developed that build upon individual, family, and community capacity and strengths, with a proactive (rather than a reactive) focus, inclusive of evaluation frameworks aligned with Indigenous concepts of wellbeing.^68^

For instance, several studies have examined the essential elements of culturally responsive residential rehabilitation services, which promote recovery and sobriety from alcohol.^69^ Enablers of success included a designated shared space for staff and residents to eat together to build trust, cohesion, and a sense of community.^69^ Furthermore, effective recovery and healing achieved in residential rehabilitation services considers alcohol dependence as a spiritual disease associated with colonialism and cultural loss.^69^ Unhealthy alcohol consumption was also considered within the construct of powerlessness associated with the interrelated factors of low education and poverty, derived from the persisting negative impacts of colonisation.^69^ Effective treatments included culturally specific job training, embracing sovereign languages, and cooking classes.^69^ However, resources were lacking to enable the effective follow-up of residents on discharge.^69^

Further research examined the role and value of culture, healing, emotional empowerment, and psycho-social wellbeing, over a 16-week program at Oolong House in New South Wales.^70^ This program provided a modified therapeutic community including evidence-based treatments such as cognitive behavioural therapy and group interventions aligned with Narcotics Anonymous and Alcoholics Anonymous techniques.^70^ Whilst these interventions formed the foundation of rehabilitation, Oolong House also promoted recovery through traditional culture and wellbeing using a holistic community-healing model.^70^ This model provided participants with structured education and activities ‘in the areas of ancestry, cultural respect, land and humanity, hunting and gathering, language, storytelling, cultural identity, traditional artwork, construction of traditional musical instruments and weapons, traditional music, cultural dance, and visiting culturally significant sites.^70^ ^(p973)^ Despite a high attrition rate, an evaluation of the program provided evidence that it led to improvements in self-efficacy in refusing alcohol, decreases in psychological distress, and feelings of emotional empowerment.^70^ Further analysis found that ‘Healing and Enabling Growth, Self-Capacity, and Connection and Purpose’ were key factors in recovery.^70^ ^(p979)^

The value of culture on Country was also qualitatively evaluated within a healing model of care to promote effective rehabilitation.^71^ Orana Haven Drug and Alcohol Residential Rehabilitation Service is a 3-month program that combines 12-Step treatment with a Therapeutic Community.^71^ An exploration of lived experiences were examined to determine people’s perceptions of the residential treatment facility.^71^ Five themes included: healing through culture and Country, emotional safety and relationships, strengthening life skills and improved wellbeing, and perceived treatment gaps.^71^ The information collected through semi-structured interviews provided evidence that the strength of the program was the value and presence of embedded culture by being on Country that enhanced connection, identity, and spirituality.^71^

## Conclusion

The devastating impact of colonisation and ongoing racially discriminatory government policies has left a trail of destruction, such that in the present day a predatory alcohol industry continues to capitalise on traumatised Indigenous Australian communities and exacerbate risky drinking behaviour. Whilst a place may exist for AMPs to reduce alcohol-related harms in some Indigenous communities, prohibition alone will not address the underlying social determinants of health and racialised health and justice systems, which impede holistic wellbeing for Indigenous Australians and their communities. Until the complex underlying issues are addressed in conjunction with harmful alcohol use, future policy making presents as an ineffective superficial band-aid that is unable to mend or heal broken lives impacted by alcohol dependence. Indigenous-led and sustainable social change needs to incorporate a holistic view of community, as demonstrated within the Interplay Wellbeing Framework. Such an approach acknowledges two-way respectful partnerships with Indigenous Australians and the value of Country, culture, healing, empowerment, and self-determination to address alcohol-related harm.

## Contributors

LH drafted the manuscript. All other authors contributed to editing and revisions. All authors have approved the final manuscript for submission.

## Declaration of interests

Lorelle Holland would like to acknowledge the financial support provided to her as a recipient of a National Health and Medical research Council (NHMRC) Post Graduate Scholarship (PGS) and the Australian Academy of Science Douglas and Lola Douglas Scholarship in Medical Science during her PhD studies (Grant number 2014148). We declare no conflicts of interest in the writing or content of the manuscript.
